# Intimate partner violence during pregnancy against adolescents in sub-Saharan Africa: a systematic review

**DOI:** 10.1136/ip-2023-044985

**Published:** 2024-01-09

**Authors:** Caroline Adjimi Nyemgah, Meghna Ranganathan, Heidi Stöckl

**Affiliations:** 1 Global Health and Development, London School of Hygiene & Tropical Medicine, London, UK; 2 Department of Global Health and Development, Faculty of Public Health and Policy, London School of Hygiene & Tropical Medicine, London, UK; 3 Institute for Medical Information Processing, Biometry and Epidemiology, Faculty of Medicine, Ludwig-Maximilians-Universität München, München, Bayern, Germany

**Keywords:** Violence, Public Health, Mental Health, Adolescent, Sexual abuse, Psychological

## Abstract

**Background:**

Adolescent pregnancy and intimate partner violence (IPV) are major public health issues that are linked to poor health outcomes particularly during pregnancy. In sub-Saharan Africa (SSA), previous studies on IPV during pregnancy have primarily focused on adults. This review examines the available evidence on adolescents’ experience of IPV during pregnancy in SSA.

**Design:**

Systematic review.

**Methods:**

We searched multiple databases for articles that met our inclusion criteria. Included studies investigated IPV during pregnancy, including prevalence, risk factors and health outcomes among ever-pregnant adolescents aged 10–19 years old or younger in SSA. Studies were peer-reviewed studies from SSA, quantitative and/or qualitative; and published in English regardless of the year of publication.

**Results:**

Nine studies out of 570 abstracts screened, published between 2007 and 2020, met the inclusion criteria. The prevalence of IPV during pregnancy among adolescents in SSA ranged from 8.3% to 41%. Mental health symptoms, particularly depression, and anxiety, were associated with adolescent IPV during pregnancy and qualitatively linked to poor coping strategies when dealing with IPV.

**Conclusion:**

This review found evidence of a linkage between pregnancy and IPV during pregnancy among adolescents. Given the long-term negative effects of IPV during pregnancy on adolescents and children, this conclusion points to the critical need for developing interventions to improve IPV detection during pregnancy in SSA among adolescents to interrupt its continuation into adulthood.

**WHAT IS ALREADY KNOWN ON THIS TOPIC**

- Adolescent mothers seem more likely than adult women to experience intimate partner violence during pregnancy with significant adverse outcomes on both the mother's and the fetus’s health.

**WHAT THIS STUDY ADDS**

- There is a paucity of quantitative and qualitative evidence on intimate partner violence during pregnancy among adolescents in sub-Saharan Africa.

**HOW THIS STUDY MIGHT AFFECT RESEARCH, PRACTICE OR POLICY**

- This paper offers evidence to help develop strategies and interventions for antenatal care to prevent physical and psychological violence-related injuries impacting the well-being of adolescent mothers and children

## Introduction

Physical and sexual violence against women by intimate partners has been recognised as a global public health concern. In 2018, the global, regional and national estimates for intimate partner violence (IPV) revealed that 27% of ever-married/partnered women between 15 and 49 experienced IPV.^1^ Among adolescents, research by Sardhina *et al* (2021) showed a higher prevalence of IPV among adolescents ranging between 21% and 28%, with sub-Saharan Africa (SSA) being among the most affected.^2^ There is also evidence on the high rate of IPV during pregnancy.^3^ Within SSA, the prevalence of IPV during pregnancy among women of reproductive age (15–49) varies and has been inconsistently reported among adolescents aged 15–19.^4 5^

Adolescents and young women in low-income and middle-income countries experience IPV at a higher rate than adults, and during pregnancy, it is even likely to be higher.^6^ A cross-sectional multicountry study that explored IPV among adolescents in vulnerable urban areas of five countries, including Baltimore, Delhi, Ibadan, Johannesburg and Shanghai revealed that Johannesburg had the highest prevalence of IPV (36.6%), with pregnancy being one of the main reasons cited by adolescents experiencing violence by their partners.^6^ Several risk factors have also been associated with adolescents’ experiences of IPV in pregnancy in SSA, including women’s low level of education, early marriage, substance abuse, childhood trauma, unemployment status, multiple sexual partners and marital status including being single or unmarried.^7–10^ Forced marriage and poverty have also found to been associated with IPV and pregnancy termination among adolescent girls and young women in 25 SSA countries,^7^ create an opportunity for partners to abuse the adolescents using different forms of IPV, including physical, sexual and emotional abuse.^7^

Moreover, other maternal factors, including age and the experience of IPV, have all been associated with neonatal complications and adverse outcomes. For instance, the fetus is directly affected by the mother’s exposure to violence during this time,^8^ increasing the likelihood of adverse maternal and child health outcomes, such as preterm and low birth weight.^11–13^ Adolescents are already more likely to develop complications, such as asphyxia and neonatal death, than older women during pregnancy, even without experiencing IPV.^14^ From the mental health standpoint, research done in South Africa examining the effect of IPV’s on mental health among pregnant adolescents between 14 and 21 years of age reported higher levels of depression, anxiety and prenatal distress among adolescents who reported IPV during pregnancy.^11^

Pregnancy comes with pressures such as lower energy, strength reduction and other physical and emotional changes.^12^ Some of the demands during pregnancy include relationship management and financial resources to manage the pregnancy. Unfortunately, for many adolescents, the lack of means to fulfil those pregnancy demands exposes them to an increased likelihood of experiencing IPV.^8 10 15^ This systematic review aims to provide evidence on the prevalence of IPV during pregnancy, coping strategies, community response and mental health outcomes associated with IPV during pregnancy among adolescents in SSA. This is important as adolescence is a crucial time for laying the groundwork for quality of life and future health programmes.^16 17^

## Materials and methods

### Inclusion and exclusion criteria

The inclusion criteria are: (1) studies focused on adolescents (aged 10–19 years) who have experienced physical, sexual and psychological violence during pregnancy; (2) studies from SSA; (3) quantitative and qualitative peer-reviewed studies with data collected from pregnant or ever-pregnant adolescents; (4) studies published in English in a scientific peer-reviewed journal between 2000 and 2021. We excluded reviews, letters, editorials, dissertations, books, studies conducted in humanitarian contexts and non-English studies. The search was conducted until February 2022, and the review was registered with the Prospective Registry of Systematic Reviews (PROSPERO) number CRD42022320144.

### Search strategy

Five health and social science databases were systematically searched to obtain studies: Ovid Medline, CINAHL, Web of Science, Cochrane Library and Africa Wide. Independently, two reviewers extracted articles and screened abstracts after they were imported into Rayyan,^18^ a systematic review management platform. After resolving any discrepancies, an extraction sheet was created in Excel to extract the information for quantitative and qualitative articles. The following search terms were used: domestic violence, spouse abuse, IPV, physical abuse, sex offence/s, rape, IPV, spousal abuse or dating violence, pregnancy, gravidity, pregnancy in adolescence, pregnancy outcome, unplanned pregnancy, unwanted pregnancy, SSA, Africa south of the Sahara, adolescent, teen, teenager and underage (online supplemental appendix A: Search terms).

10.1136/ip-2023-044985.supp1 Supplementary data

**Figure 1:**
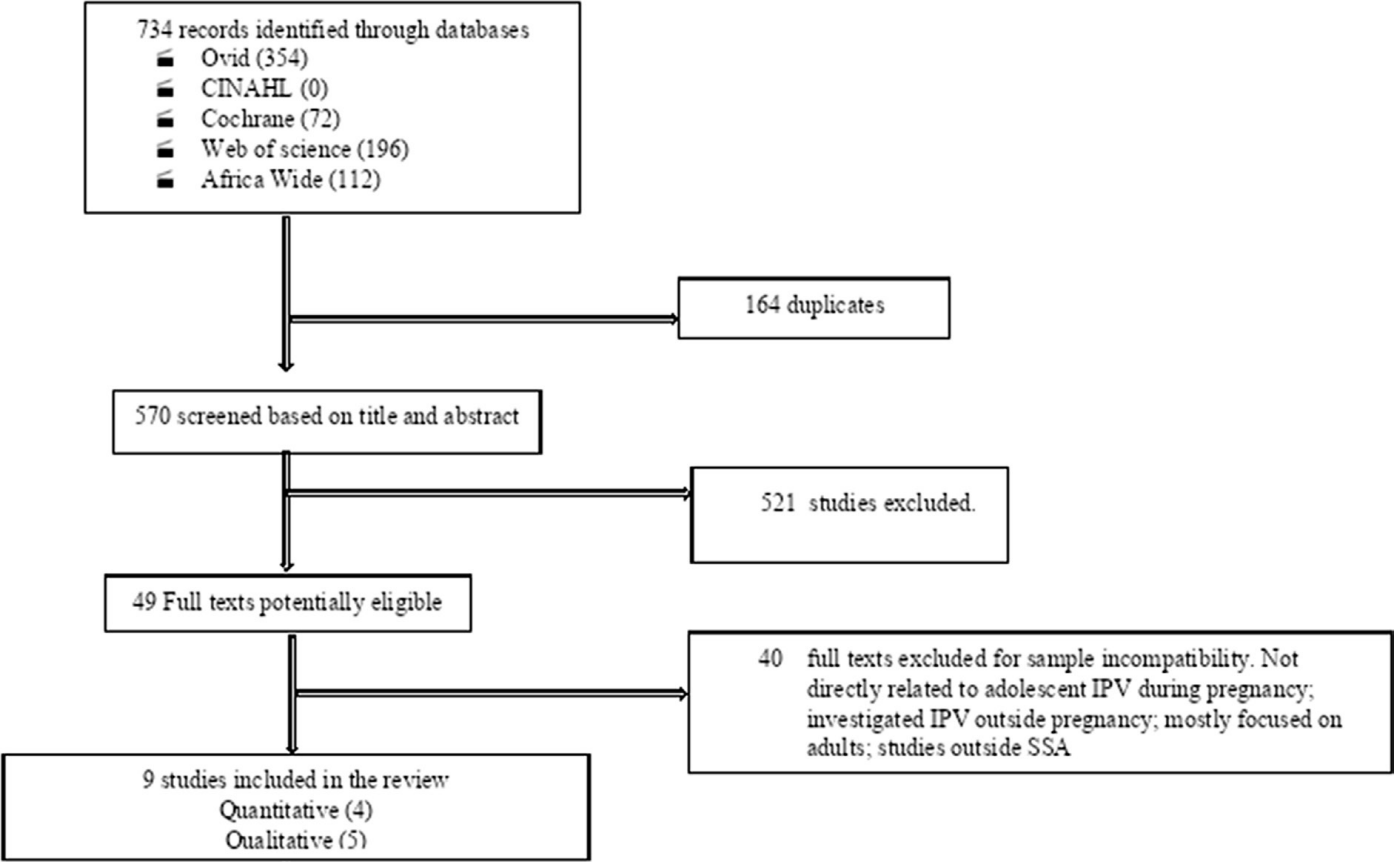
Preferred Reporting Items for Systematic Reviews and Meta-Analyses extraction flow chart illustrating the process of identifying included studies.

The results yielded 734 potential studies. The first author (CA) and a second reviewer (CL) screened the 734 abstracts, during which 164 duplicates were removed, yielding 570 abstracts. The 570 abstracts were screened against the inclusion criteria to determine their eligibility. This process produced 49 potentially eligible articles. After a full-text review against the inclusion criteria, nine articles were selected. The articles were discussed in detail with other authors (HS and MR) to ensure rigorous and critical data analysis. Figure 1 describes within a Preferred Reporting Items for Systematic Reviews and Meta-Analyses flow chart the overall identification process of the studies included in this systematic review.

### Study selection and data extraction

A pre-established framework created for the research in Excel was used to extract data from the chosen studies. Data were extracted and organised into the following categories: author, setting, sample size, study type, study design, measures, quality appraisal and study outcomes.

### Quality assessment

Quality appraisal of cross-sectional studies was conducted using the Joanna Briggs Institute Critical Appraisal Skills checklist for studies reporting prevalence data,^19^ while the Critical Appraisal Skills Programme checklist (CASP),^20^ was adapted to assess qualitative studies. The checklist for studies reporting prevalence was a nine-item questionnaire grouped into categories to evaluate the sample, study design, the possibility of bias and data analysis. Qualitative studies were reviewed by adopting 10 questions assessing the number of participants, design and analysis. A question was asked initially on four scales, including ‘Yes,’ ‘Can't tell’ and ‘No’. The questionnaire was readapted as follows to meet our study objectives on adolescent pregnancy and IPV ‘Yes=1, No=0, Unclear=0, Not applicable=0’. Three questions were added to the CASP as a guide to the reviewers to determine whether the papers directly investigated issues related to IPV during pregnancy among adolescents in SSA.

According to the checklist, a combined score between six and nine indicates high quality, whereas zero to five indicates low quality. The assessment score of the first author was carefully reviewed and discussed with coauthors following a conclusive quality assessment decision. The quality of all qualitative studies was deemed satisfactory, with an average score of 8. On the other hand, quantitative studies were judged to be of low-quality ranging between 2 and 5. However, no study was excluded from the review because of unsatisfactory quality.

### Patient and public involvement

Patients or the public were not involved in the design, or conduct, or reporting, or dissemination plans of our research as this is a systematic review.

## Result

Table 1 highlights the main findings of studies investigating IPV in adolescent pregnancies. Nine studies were identified all conducted in SSA and published between 2007 and 2020: three in Uganda,^15 16 21^ two in South Africa,^17 22^ two in Ethiopia,^23 24^ and one study each in Kenya,^25^ and Nigeria.^26^ Five studies were done in health facilities, and the remaining four in communities. The sample size varied from 15 to 61 adolescents for qualitative studies and 29–61 in quantitative studies. The adolescents’ age ranged from 10 to 19 years.

### Study characteristics

The four quantitative studies used cross-sectional designs,^22–24 26^ three qualitative studies used focus group discussions,^15 17 25^ and the other two qualitative studies used in-depth interviews.^16 21^ Eight studies collected data specifically on adolescent experiences of partner violence during pregnancy. One of the studies focused on women of reproductive age in general, but because it contained useful information on adolescents, it was included.^26^ IPV was measured differently: two studies used the WHO multicountry study on women’s health and domestic violence against women questionnaire, one used the WHO modified conflict tactics scale on physical and psychological violence, and one used a self-developed semistructured questionnaire, whereas four qualitative studies used semistructured interview guidelines and one abuse assessment screen questionnaire. Overall, all the quantitative studies focused on physical, sexual and/or emotional IPV, whereas qualitative studies focused on physical or emotional IPV as shown in the (online supplemental table 1). The quantitative findings were reported in percentages, odd ratios, p values, and CIs and the qualitative studies reported key themes related to adolescent experiences of IPV during pregnancy.

### Study outcomes

The quantitative studies reported on prevalence and health outcomes with sample sizes ranging from 15 to 61 adolescents. The prevalence of IPV during pregnancy ranged from 8.3% in Nigeria,^26^ to 41% in South Africa.^22^ The two studies in Ethiopia in 2019 and 2020 reported a prevalence of 17.1% and 37.9%.^23 24^ Regarding evidence on IPV during pregnancy and health outcomes, the systematic review only found and reported associations with sexually transmitted infections, and depression. A cross-sectional study of IPV experience during pregnancy in South Africa showed an association between IPV and postpartum sexually transmitted infections in 61 adolescent mothers (RR: 4.32; 95% CI 0.95 to 19.69). 13.1% of adolescents who reported IPV during pregnancy were diagnosed with at least one sexually transmitted infections (gonorrhoea, chlamydia, trichomonas) compared with those who did not report any IPV during pregnancy even after controlling for covariates (aRR: 4.43; 95% CI 1.31 to 14.97).^22^ In Ethiopia, maternal depression was found to be associated with IPV during pregnancy with 17.7% of adolescents reporting experiencing IPV while pregnant and also reporting being depressed.^24^

Furthermore, we noted that the only health outcomes investigated in the quantitative studies captured only sexually transmitted infections and depression, while a much wider range of health outcomes are known to be associated with violence during pregnancy among women of reproductive age. Future studies on violence during pregnancy among adolescents need to explore a wider range of health outcomes

There were three salient overarching themes that emerged from the qualitative studies and are presented below. These are: (1) partner denial of pregnancy responsibility and controlling behaviours, (2) adolescents coping mechanisms to adapt and survive physical and psychological violence by partner, and (3) partner and community responses to adolescent pregnancies.

#### Theme 1: partner denial of pregnancy responsibility and controlling behaviours

Included qualitative studies reported that IPV during pregnancy is inflicted by partners who deny pregnancy responsibility and exercise pressure on adolescents to terminate the pregnancy through physical violence. In Uganda, 15 pregnant adolescent mothers reported physical violence, including beating leading to hospitalisation and bleeding inflicted by partners who denied pregnancy responsibilities.^21^ Similar findings were reported among adolescent mothers in Kenya,^25^ where male spouses threatened to leave or demanded that the adolescent mother terminate her unplanned pregnancy. The persistent quarrels were exacerbated by substance abuse by the male partner, acceptance of IPV and the stigma displayed in the community. Adolescents also expressed worries regarding paternity and the mindset imposed on them by the situation. This situation exposes them to elevated stress levels, suicidal ideation and depression due to their partners’ denial of pregnancy responsibility.^25^

#### Theme 2: coping strategies used by adolescents to deal with IPV during pregnancy

As described by Kaye *et al* in their study examining adolescents coping mechanisms within the context of IPV during pregnancy,^15^ withdrawal of partners was a common strategy used by pregnant adolescents, yet one that rarely succeeded. Pregnant adolescents found themselves unable to detach themselves from the love and emotional attachment they had with the abuser and his economic support, and ultimately leave. One of the participants discusssed how despite the violence it was difficult to leave because she does not know how to look after herself when pregnant without him, so remains hopeful that he will change. In the same study, many respondents reported using any excuse and available opportunity to leave their partners temporarily. Some adolescents used retaliation or fought back in response to the violence. This included informing the police or local council leaders to have the spouse penalised or reprimanded.

#### Theme 3: partner and community responses to adolescent pregnancies

Adolescent mothers mentioned that while sometimes favourably, most often negatively, the news of their pregnancy changed their relationships with their partners. Rejection and rage were the unfavourable responses. In South Africa, one study found that adolescent pregnancy can cause physical and emotional violence by partners and community members. Exploring the experiences among a sample of 18 adolescent parenting mothers from KwaZulu-Natal, the relationship with the father of the child and the father–child interaction emerged as complex. The group discussions revealed adverse reactions to the unplanned pregnancy by partners, including denial of responsibility, abuse through vulgar language, public humiliation, aggressiveness, cheating and lack of attention throughout the pregnancy.^17^

Adolescent girls or young women who became pregnant while unmarried faced severe repercussions, including stigma and social isolation, within the community, in schools, homes and from male partners, fostering negative sentiments such as abortion towards the pregnancy.^16^ Another study in Uganda exploring the pregnancy experience of 26 adolescents in the Rakai community cohort study found similar results.^16^ A 7-year-old participant spoke about the stigma encountered by those who chose to have an abortion.

Although several coping strategies were used to seek help, the outreach often worsened IPV.^15^ Reaching out to friends, family and local leaders was another coping mechanism used by expectant adolescents. These individuals could step in and stop the attack by reprimanding the partner. However, in some cases, it worsened the situation for adolescent mothers because it exacerbated domestic violence, even though it was occasionally of great assistance to some participants. Some respondents advised against it and named it one of the things that made their reputation worse. For example, when one woman explained that she reported her partner to the local chairman, she could see a positive effect on him, yet one that only lasted for a few days.

## Discussion

To our knowledge, this is the first systematic review of IPV during adolescent pregnancy in SSA. This study examined available knowledge from nine studies of adolescent experiences of IPV during pregnancy in five SSA countries. The prevalence of IPV during pregnancy among adolescents ranged from 8.3% to 41%. Yet, these rates are not directly comparable due to different study designs, settings and populations. The findings suggest an association between adolescent IPV during pregnancy and antenatal depression. A direct association between IPV during pregnancy and depressive symptoms has been well researched in adults compared with adolescent-related studies.^27^

Women of reproductive age (15–49) have been found vulnerable to mental issues, with a peak during pregnancy.^28^ In Ethiopia, a survey of women of reproductive age showed a consistently higher prevalence of depression during the antenatal period among adolescents compared with other age groups.^24^ In the current review, the type of IPV associated with depressive symptoms included physical, sexual and psychological violence,^24^ which is consistent with those found in women of reproductive age in India,^29^ and Bangladesh,^30^ where physical and sexual IPV were more likely to be associated with depressive symptoms during pregnancy. Women who reported emotional, physical and sexual IPV were also more likely to be diagnosed with depression. Due to adolescents lacking basic life skills, such as problem-solving and decision-making, this can affect their ability to handle stressful events,^15^ thus exposure to physical IPV during pregnancy may be more likely to lead to depression among adolescents compared with older women, but further research is needed. Nonetheless, the impact of IPV on depressive symptoms can be affected by a number of variables, including the severity and frequency of the violence, the timing of the violence during pregnancy, and the social support that the individuals have access to. Since adolescent pregnant women have special experiences, it is therefore important to carefully analyse the individual nuances of these connections even though there may be similarities in the association between IPV types and depressive symptoms across age groups. Due to adolescents lacking life skills for problem-solving and decision-making that can affect their appraisal of stressful events,^15^ exposure to physical IPV during pregnancy may be more likely to lead to depression among adolescents compared with older women, but further research is needed.

The qualitative studies also showed different ways adolescents cope with partner violence. These coping strategies include withdrawal, retaliation and help-seeking. These coping strategies are similar to research on adults dealing with IPV from Uganda.^15^ Adults coping strategies within a power relationship context, divided into four stages: the binding stage, the enduring stage, the disengagement stage and the recovery stage, carry some similarities with those of adolescents. However, these stages have been defined from a problem-focused viewpoint of adults, while adolescents are emotionally focused.^15^ Violence during pregnancy by a partner, paired with rejection and denial of pregnancy responsibility, prompted adolescents to isolate themselves, increasing the risk for mental health problems and decreasing their self-esteem and sense of hopelessness.^15^ Our findings showed that denial of pregnancy responsibilities and pressure on adolescents to terminate pregnancies increased the risk of experiencing different types of IPV, leading to severe and mild consequences such as beating and bleeding. Both partners and community members have been found to encourage and reinforce IPV during adolescent pregnancy by not taking appropriate action to deal with the complaints. Unfortunately, the lack of action from community members or stakeholders and the adolescents’ inability to decide on their reproductive health appears to increase the chance of experiencing IPV during pregnancy.^31^

### Strengths and limitations

The comprehensive synthesis of prior research by this systematic review on IPV during adolescent pregnancy provides gives a clearer picture of the current level of knowledge in this research space. It presents this strong viewpoint on the subject by incorporating studies of various quality levels and by using a mixed-methods methodology. Additionally, because it shines light on a vulnerable population group and emphasises the need for focused interventions and assistance, the review’s focus on adolescent pregnant women is particularly pertinent. Although this review is only based on a small sample of adolescent studies and cannot be conclusive, it presents consistent information across countries to indicate that pregnancy does not appear to protect adolescents from IPV. This current review and the focus on SSA are believed to be of great interest and positive impact as it provides information about potentially important predictors and health outcomes for planning prevention work in antenatal care and implementing new programmes and routines to identify adolescents who experienced partner violence during pregnancy in SSA.

Nevertheless, our review revealed crucial gaps in knowledge. To date, the evidence on IPV during adolescent pregnancy is limited to only nine small-scale studies. Due to the limited empirical evidence related to IPV during adolescent pregnancy in SSA and the focus only on specific countries, the findings may not be generalisable to all adolescents living in SSA. Adding to that, the low sample size of the individual quantitative studies included in the review present a serious limitation. The underlying reasons for the small sample sizes were due to logistical limitations and the complex nature of the subject. While these studies offer valuable insights into the experiences of adolescent mothers, the limited sample sizes compromise the overall generalisability of the systematic review and results have to be interpreted accordingly.

To only include empirical articles, the search was limited to peer-reviewed papers, excluding evidence related to IPV during pregnancy among adolescents in SSA, such as book chapters and WHO reports. This study excluded any study done in a language other than English, thus decreasing the chance of inclusion for studies done in non-English-speaking SSA countries. Finally, most studies were done in East Africa, with only one study in West Africa. Few studies report on risk and protective factors, as most focused on the prevalence and health outcomes. Therefore, future studies must investigate the drivers leading to IPV during adolescent pregnancy and explore family dynamics to unravel more clues to uncover the causes of IPV during pregnancy to propose research-oriented solutions and policy recommendations. As very few studies focused on interventions for IPV during pregnancy, there is an urgent need for more research studies that should suggest appropriate interventions for each setting since social norms significantly influence the approval and disapproval of IPV.^32^ In order to establish an adolescent-focused theories from which treatments to reduce IPV during pregnancy would be derived, it is necessary to strengthen IPV-related theories, such as sociological theory.

Using a socioecological lens could provide essential insights into the larger societal dynamics, power relationships and cultural norms influencing IPV prevalence and persistence among this vulnerable population. Incorporating socioecological lens can explore how age, gender, socialisation and power dynamics intersect to shape the experiences of pregnant adolescents because adolescence is a time of dynamic social transformations and identity construction. This will give a detailed insight of the impact of sociocultural factors on the prevalence and consequences of IPV, allowing for the development of interventions to support young mothers and subvert harmful norms. Having a better understanding of the socioecological factors influencing IPV during adolescent pregnancy can help build successful prevention initiatives and policy recommendations.

It is crucial to highlight that adding studies with lower quality, as is the case with most quantitative studies included in this review. We have included them given the general dearth of quantitative studies on this issue, and to highlight that even those that exist are not of good quality, calling for an increase of quantitative studies on the subject. Given the patchy evidence base, even the low-quality studies unfortunately are the only evidence based we can have to understand IPV during pregnancy in adolescents in SSA.

## Conclusion

In response to the lack of research on IPV during pregnancy among adolescents in SSA, we systematically reviewed empirical studies to synthesise the evidence of IPV during pregnancy and its health consequences. Although some studies showed valuable information regarding adolescents’ experiences of IPV during pregnancy, it is difficult to conclude that adolescents are more likely to experience IPV than adults; therefore, more studies should investigate the prevalence, severity, drivers, mechanisms and interventions that can intervene early before adolescents enter relationships. We hope this research will be considered as a basis for developing future research questions and hypotheses that will deepen our understanding of adolescents’ experiences of IPV during pregnancy and move towards more evidence-based practices and adolescent-focused interventions.

## Data Availability

All data relevant to the study are included in the article or uploaded as supplementary information.
