# Supplementary material for: Intimate partner violence during pregnancy against adolescents in sub-Saharan Africa: a systematic review
Source: Inj Prev. 2024 Jan 9;30(3):177–82. doi: 10.1136/ip-2023-044985 (PMC11137446; doi:10.1136/ip-2023-044985)
Supplement: Supplementary data [file ip-2023-044985supp001.pdf]

<u>Appendix A:</u> Search terms

#	Search terms	Hits
1	domestic violence/ or spouse abuse/ or intimate partner violence/ or physical abuse/ or rape/sex offenses/ or rape/(intimate partner violence or IPV or spouse abuse or dating violence).mp. [mp=title, abstract, original title, name of substance word, subject heading word, floating sub-heading word, keyword heading word, organism supplementary conce word, protocol supplementary concept word, rare disease supplementary concept word, unique identifier, synonyms] (sexual abuse or physical abuse or gender-based violence or gender based-abuse or emotional abuse or emotional violence).mp. [mp=title, abstract, original title, name of substance word, subject heading word, floating sub-heading word, keyword heading word, organism supplementary concept word, protocol supplementary concept word, rare disease supplementary concept word, unique identifier, synonyms]	49950
2	pregnancy/ or gravidity/ or pregnancy in adolescence/ or exp pregnancy outcome/ or pregnancy, high-risk/ or pregnancy maintenance/ or pregnancy, unplanned/ or pregnancy, unwanted/(pregnan* or obstetric* or labor or maternity).mp. [mp=title, abstract, original title, name of substance word, subject heading word, floating sub-heading word, keyword heading word, organism supplementary concept word, protocol supplementary concept word, rare disease supplementary concept word, unique identifier, synonyms] (abortion* or fetus mortality or fetal mortality or miscarriage*).mp. [mp=title, abstract, original title, name of substance word, subject heading word, floating sub-heading word, keyword heading word, organism supplementary concept word, protocol supplementary concept word, rare disease supplementary concept word, unique identifier, synonyms] exp Abortion, Induced/ exp Abortion, Threatened/ or exp Abortion, Spontaneous/	1139020
3	1 and 2	4809
4	(sub-saharan africa or subsaharan africa or africa south of the sahara).mp. [mp=title, abstract, original title, name of substance word, subject heading word, floating sub-heading word, keyword heading word, organism supplementary concept word, protocol supplementary concept word, rare disease supplementary concept word, unique identifier, synonyms] exp "africa south of the sahara"/ or exp africa, central/ or exp africa, eastern/ or exp africa, southern/ or exp africa, western/	228830
5	3 and 4	549
6	(adolescen* or teen or teens or teenager* or young wom?n or under-age* or underrage).mp. [mp=title, abstract, original title, name of substance word, subject heading word, floating sub-heading word, keyword heading word, organism supplementary concept word, protocol supplementary concept word, rare disease supplementary concept word, unique identifier, synonyms] adolescent/ or young adult/exp adolescent behavior/ or exp underage drinking/youth*.mp. [mp=title, abstract, original title, name of substance word, subject heading word, floating sub-heading word, keyword heading word, organism supplementary concept word, protocol supplementary concept word, rare disease supplementary concept word, unique identifier, synonyms]	2624461
7	5 and 6	354
